# Photovoltaics: Reviewing the European Feed-in-Tariffs and Changing PV Efficiencies and Costs

**DOI:** 10.1155/2014/404913

**Published:** 2014-05-14

**Authors:** H. L. Zhang, T. Van Gerven, J. Baeyens, J. Degrève

**Affiliations:** ^1^Department of Chemical Engineering, Chemical and Biochemical Process Technology and Control, Katholieke Universiteit Leuven, 3001 Heverlee, Belgium; ^2^Department of Chemical Engineering, Process Engineering for Sustainable Systems, Katholieke Universiteit Leuven, 3001 Heverlee, Belgium; ^3^School of Engineering, University of Warwick, Coventry CV4 7AL, UK

## Abstract

Feed-in-Tariff (FiT) mechanisms have been important in boosting renewable energy, by providing a long-term guaranteed subsidy of the kWh-price, thus mitigating investment risks and enhancing the contribution of sustainable electricity. By ongoing PV development, the contribution of solar power increases exponentially. Within this significant potential, it is important for investors, operators, and scientists alike to provide answers to different questions related to subsidies, PV efficiencies and costs. The present paper therefore (i) briefly reviews the mechanisms, advantages, and evolution of FiT; (ii) describes the developments of PV, (iii) applies a comprehensive literature-based model for the solar irradiation to predict the PV solar energy potential in some target European countries, whilst comparing output predictions with the monthly measured electricity generation of a 57 m² photovoltaic system (Belgium); and finally (iv) predicts the levelized cost of energy (LCOE) in terms of investment and efficiency, providing LCOE values between 0.149 and 0.313 €/kWh, as function of the overall process efficiency and cost. The findings clearly demonstrate the potential of PV energy in Europe, where FiT can be considerably reduced or even be eliminated in the near future.

## 1. Introduction

Following a strong public concern, conventional (nuclear and coal) electricity generation projects have recently been postponed, highlighting the negative perception towards these generation technologies and their environmental impact. These events also generated uncertainty in the electricity market, whilst creating a very favorable context towards new renewable energy projects, with a focus on using solar energy [1–4]. The most significant renewable energy production is provided by wind and solar power, with annual growth rates exceeding 30% over the past years [5–7]. The importance of this subsector of renewable energy is witnessed by the exponential growth of the number of publications. Reports for PV include both general applications (e.g., [8, 9]) and fundamental aspects (e.g., [10–12]). Research on wind energy also covers specific applications (e.g., [13, 14]), whilst also environmental and costing parameters are frequently reported in [6, 15–18]. Biomass will also play a key role in the future (e.g., [19–21]). The potential of hydroelectricity is limited to some distinct regions in the world.

**Figure 1:**
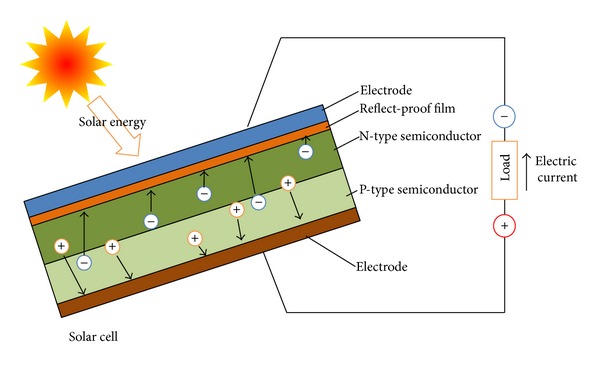
Schematic operating principle of a PV solar cell (adapted from [22]).

By ongoing PV development, with higher transformation efficiencies of solar power to electricity and cheaper costs of PV modules, the European contribution of solar power increases exponentially from over 100 GW in 2012, with an annual minimum growth of 40 GW during the coming years. In March 2007, the European Union targeted 20% renewable energy for 2020, with special emphasis on small scale units [24]. To enhance the rate of this development, it is necessary to update the insights, the tools, and the technical/economic analysis. Within the solar energy technologies, photovoltaics (PV), to a large extent, and concentrated solar power technology, to a lesser extent, have been widely investigated and applied in many European countries. PV draws a significant focus, with a guaranteed future in view of the ongoing developments. Solar cells, mostly made out of semiconductors, have been used since the 1950s for absorbing solar energy and converting it directly into electrical current: the semiconductor material captures photons emanating from the sun, and the absorbed photons create an electron-hole pair. The movement of billions of these electrons in the same direction under an internal electric field creates a current. The internal field is formed by the combination of materials with positive charges (p-type) and negative charges (n-type). A number of solar cells compose a solar module which can be used as an electricity generator. The fundamental mechanism of the different semiconductors has been dealt with in detail in numerous papers (e.g., [22, 25]) and is schematically represented in Figure 1.

Photovoltaic materials presently used include monocrystalline, polycrystalline, and amorphous silicon, cadmium telluride, and copper indium/gallium selenide/sulfide. The manufacturing of solar cells and photovoltaic arrays has advanced considerably in recent years, and new forms of PV, such as thin-film solar cells and concentrators, have been developed [22, 26–31]. More than 100 countries use solar PV. Installations are mostly ground-mounted or built onto the roof or walls of a building (building-integrated or rooftop). With an ongoing “revolution” in photovoltaics by higher transformation efficiencies of solar power to electricity and cheaper costs of photovoltaic cells, the contribution of solar power has increased exponentially, optimistically capable of meeting most of the electricity demands by about 2030 [3].

For investors, operators, and scientists, it is important to know how much electricity will be generated by the PV system and how well the PV system will perform.

To provide an updated insight into these questions, the present paper will (i) briefly review the mechanisms, advantages, and evolution of feed-in-Tariffs (FiTs), (ii) describe the revolutionary developments of photovoltaics, (iii) apply a comprehensive literature-derived model for the solar irradiation to predict the PV solar energy potential in some target countries, (iv) assess the monthly measured electricity generation by a 57 m² photovoltaic system (Belgium) in terms of productivity, and finally (v) predict the levelized cost of energy (LCOE) of photovoltaics in terms of varying investment and efficiency.

PV modules are commonly characterized by their Wp (Watt-peak) equivalent, being a measure of the nominal power of the PV module by determining the current and voltage while varying the resistance under defined laboratory illumination, at 1 kW/m² and 25°C. This peak power value serves a reference and is given per m² area of the PV module.

The annual total of global irradiation, *H*, that hits the module, is specific for each location and should be obtained from databases, measurements, or calculations. The target yield is the total annual energy produced on the direct current (DC) side of the PV module. The actual yield is the annual produced amount of electricity delivered as alternating current (AC). The operation factor, or performance ratio, is defined as the ratio between the actual yield (annual production of AC electricity) and the target yield. It is a useful factor to compare systems and accounts for preconversion losses (*η*_pc_), module and thermal losses (*η*_mt_), and system losses (*η*_sys_).

The performance ratio, PR, is therefore given by

(1) PR=(Actual  AC⁡  Yield)(Target  DC  Yield)=EEtarget=ηpcηmtηsys=η.

The preconversion losses account for effects of shading, dirt, snow, and reflection losses that increase with the angle of incidence. Module losses include low and high energy photons not caught, recombination of photons, and other losses in the depletion zone. Thermal losses increase with increasing temperature and depend upon the location, wind speed, and mounting methods (glass, thermal properties of construction materials). System losses involve mostly wiring resistance and inverter efficiency.

Sometimes, the energy yield is defined in terms of the peak power of the module as

(2) $$\begin{array}{c} \frac{E}{E_{\text{peak}}\;}=\text{PR}\left(\frac{H^{*}}{H_{\text{ref}}}\right) \end{array}$$

with H* as annual sum of global irradiation in kW/m² and *H*_ref_ is the reference peak irradiation of 1000 kW/m². This is a very interesting ratio, since *E* determines the earnings potential, whilst *E*_peak_ reflects the size and costs of the PV system.

## 2. Feed-in-Tariff Mechanisms Boost the Use of Photovoltaics and Renewable Energy

In March 2007, the European Union targeted 20% renewable energy for 2020, with special emphasis on small scale units [24]. To instigate the renewable energy market, many European and numerous other countries, for example, USA, have implemented promoting policies, such as the feed-in-Tariff mechanisms, to boost solar PV and other renewable energies [32, 33]. A comprehensive review of FiTs is given by [34, 35]. The application in European countries illustrates the impact of the FiTs and is illustrated hereafter for a few selected and representative countries. FiTs have been an effective mechanism to kick-start the development of PV and other renewable energy sources, creating a coexisting economical market for independent power producers by a guaranteed access to the national grid and issuing “tradable green certificates” (TGC) as equivalent guarantees of power sales and investment. Other countries, for example, the Netherlands, also offer specific subsidies for private and industrial PV investors, on a tender basis. This subsidy mechanism also existed in, for example, Belgium but was abandoned since complementing TGC and no longer deemed necessary due to the price decrease of PV. These long-term financial arrangements have comforted banks in risk mitigation. The relative success of this policy in various European markets (Germany, France, Italy, and Spain) can be largely explained by the extent to which governments have succeeded in meeting two key conditions, that is, by explicitly linking FiTs policies to well-defined and binding targets that are part of a broader energy and climate policy and by providing investors with a high degree of transparency (easy to navigate through the structure), of longevity (creating a stable and durable environment), and of certainty (providing measurable and sufficient revenues to support a reasonable rate of return).

Germany firstly (1990) adopted a “law on feeding electricity into the grid” [36], requiring a guaranteed purchase of utilities from renewable energy sources at prices that were determined as a percentage of the prevailing retail price of electricity. The percentage offered to solar power was set at 90% of the residential electricity price. Similar percentage-based feed-in laws were adopted in Spain [37], as well as in Denmark [38]. Germany reframed the feed-in law in 2000 as the Act on granting priority to renewable energy sources (amended in 2004 and 2008), proceeding as the world most effective and successful policy framework at accelerating the development of renewable energy technologies: the new Act defined that new prices should account for the economy of scale and differ for wind power, solar power, biomass and biogas sources, and geothermal energy. Purchase guarantees were extended for a period of 20 years, albeit accounting for progressive reductions, in a mechanism known as “tariff degression” [39]. Driven by the policy, solar PV in Germany has resulted in a drop of peak electricity prices by up to 40% with savings between 520 million and 840 million € for consumers [40]. At the same time, the surcharge paid by electricity consumers has increased, reaching an overall cost for consumers of 20,000 million € in 2013 [41].

The Spanish feed-in legislation was set by Royal decree 1578/2008. For photovoltaic installations, the decree categorized two installations, that is, building-integrated installations and nonintegrated installations, with different power scales with differentiated Tariffs. In 2010, the Spanish government has retroactively reduced the Tariffs paid to renewable generators. However, given a series of factors, and the unsustainable cost of the FiT mechanism, the Spanish government (27/01/2012) temporarily stopped accepting new FiT applications for projects starting operation after January 2013 [42]. Over 2600 MW solar capacity was installed in Spain in 2008, exceeding the expected 400 MW.

In France, the administrative procedure for the ground-mounted PV market segment was significantly modified in the late 2009. Because of the complexity of the administrative process, the feed-in-tariff (FiT) is only attractive for installations less than 100 kWp according to the decree of 4 March 2011. Above 100 kWp, the FiT is established at 0.117 €/kWh (until 30 September 2011, with further reductions in 2012), thus severely limiting the profitability of the installation [43].

Feed-in-Tariffs in UK were first announced in October 2008, then applied by early April 2010, and modified in March 2011 to exclude large-scale photovoltaic installations (exceeding 50 kW) [44]. It is believed that the UK government tried to trim the Tariffs for large systems in order to benefit smaller domestic systems. As of April 2012, 1,452 GW PV was receiving FiT payments, with a major participation of <4 MW units [45]. Estimations show that a typical PV system costing *£*7,500 pays for itself in less than 8 years, generating *£*23,610 over 25 years. [46]. A future reduction of the FiT mechanism in the UK is moreover likely [47, 48].

Italy introduced FiT in February 2007, leading to a considerable fraction (5.6%) of the total energy needs produced by PV, increasing by an impressive 71.8% within the renewable energy pool. In December 2012, PV systems produced 749 GWh of energy providing 2.81% of the month's energy demand [49]. Italy also stopped the FiT programme in July 2013 when the subsidy hit the budget cap [50].

By 2012, FiT policies have been enacted in over 50 countries, including Belgium, where, by July 3, 2012, 1.827 MW has been registered by the Flemish regulator of the electricity and gas market [51], which is an equivalent of slightly over 2 GWp. Although not applying FiTs, the Netherlands offers subsidies for PV private and industrial investors, with an additional tax reduction for companies [52, 53].

Similarly, a very generous feed-in programme led an explosive growth of PV capacity in Czech Republic, Bulgaria, and Greece. As a consequence, the Czech parliament approved a plan to end renewable energy subsidies for new projects and imposed a 10% tax on existing solar plants in 2010 [54]. Bulgaria and Greece also introduced a tax on existing renewable energy generators to reduce the unsustainable subsidy cost [55]. Overall, these examples support some critics received by FiT that their performance depends to a large part on the cost estimation made by the regulator [56–58]. Hence, the fact that FiT mechanisms have been very effective may indicate that the Tariffs were set too high.

The boom of markets in Europe illustrates the success and future uncertainties of the FiT mechanism. Because of PV's unique characteristics (resource availability, ease of siting, modularity, and ease of installation), the markets grew too fast without a well-thought out-plan to adapt, compelling governments to adjust or unexpectedly curtail FiTs, thereby undermining investor confidence. The implementation and development of FiTs in Germany and to some extent in Italy, however, provide several useful lessons, being (i) an increasing investor confidence and reaching a minimum renewable energy target (30% in Germany by 2030); (ii) using objective and transparent measures to generate adjustments in the FiT levels; (iii) making use of automatic price adjustments to govern volumes with transparent formulas that have successfully tracked the rapid decrease in cost of PV systems; and (iv) lifting unexpected interventions only to the extent of being absolutely necessary to maintain the longevity and durability of the FiT system. Recent developments in increasing PV efficiencies and decreasing PV costs, as discussed below, will further reduce the levelized cost of energy (LCOE) and will make the further application of FiTs to PV systems unnecessary.

## 3. Predicting the Solar Irradiation Levels

The most important inputs towards PV design are the annual, monthly, and daily solar irradiation fluxes, both of direct and diffuse nature. With only monthly averages available (e.g., NASA), it is necessary to apply a methodology that converts these values into hourly databases. The underlying equations of such a methodology were presented and applied in previous papers [4, 59]: the approach updated and combined the essentials of numerous publications into expressions of daily distributions and hourly variations of solar irradiations for any selected location, starting from the monthly average solar irradiation value, and generating a sequence of daily and hourly solar irradiation values. Such a sequence represents the trend of solar irradiation in a specific area, with respect to the values observed, the monthly average value, and its distribution (the “good and bad” days). The daily total irradiation is obtained by applying the daily clearness index, *K*_*T*_, and the daily extraterrestrial irradiation *H*_0_. Considering that a PV power plant accepts a certain percentage of diffuse irradiation, the present paper considers the total (direct + diffuse) solar irradiation as energy input. The literature-based methodology by Zhang et al. [4] enables designers to calculate the solar irradiation at any specific location and provides essential data in support of PV power plant output and economical evaluation. By way of example, the following European locations (Table 1) were calculated and compared. Average weather conditions are given in Table 2.

**Figure 2:**
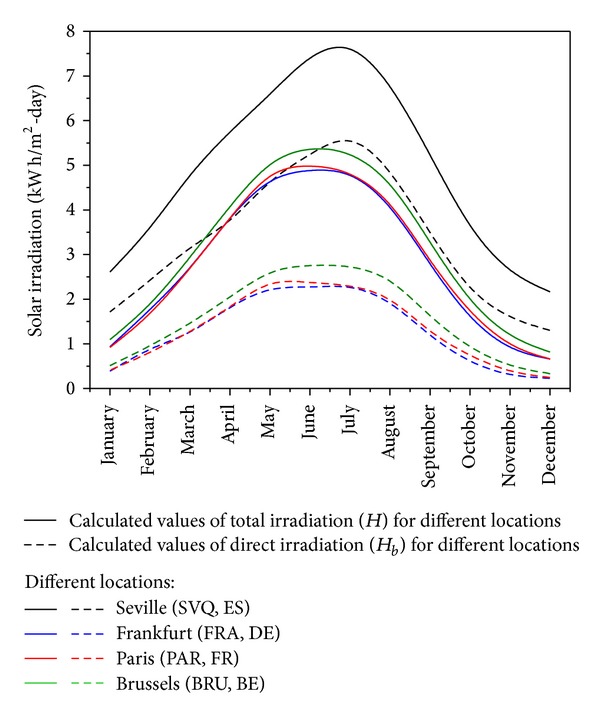
Calculated values of total (*H*) and direct (*H*_*b*_) irradiation for different locations: Seville (SVQ, ES), Frankfurt (FRA, DE), Paris (PAR, FR), and Brussels (BRU, BE) [23].

The monthly solar irradiation, *H*, is illustrated in Figure 2 for the different locations and shows the latitude and seasonal dependencies, whilst also illustrating the maximum and minimum values obtained throughout the year. The calculation shows that even a location of high latitude, for example, Brussels, has sufficient solar irradiation levels to justify the implementation of PV systems. Assuming an average transformation efficiency of 20%, the average annual power generation in Brussels is approximately 154 kWh/m^2^ in the time duration between April and September. Similar power generation levels are expected for Frankfurt and Paris, whilst Seville will score significantly better due to the higher levels of solar irradiation due to its more southern location.

**Figure 3:**
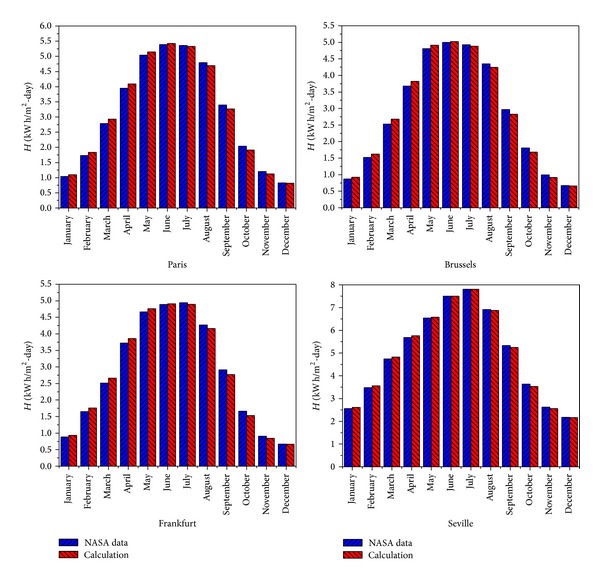
Monthly NASA solar irradiation (blue rectangle) and model-calculated data (red rectangle) for Brussels, Seville, Paris, and Frankfurt.

Comparing the model calculations with NASA-data [60], a good agreement is obtained, as shown in Figure 3. The model tends to slightly overestimate the solar irradiation *H* in the first half year, while underestimating it in the last half, with a maximum difference between model and NASA-data <8%.

**Figure 4:**
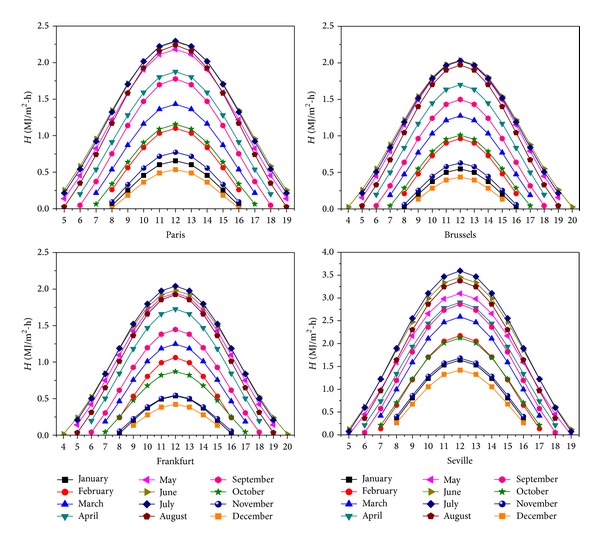
Hourly evolution at the 15th of the respective months in Paris, Brussels, Frankfurt, and Seville.

Illustration of the model prediction on an hourly basis is given for the 4 European locations in Figure 4, and this for each 15th day of the month, where the radiation flux can be seen to increase from sunrise to noon, thereafter decreasing again till sunset.

Having established the annual, monthly, and daily levels of the total solar irradiation, its impact on the power yield of the PV can now be assessed, considering the transformation efficiency of the PV cells.

## 4. The Photovoltaics Revolution in Transformation Efficiency and Costs

Driven by advances in technology and increases in manufacturing scale and sophistication, the cost of photovoltaics has declined steadily since the first solar cells were manufactured [61] and the levelized cost of energy (LCOE) from PV is gradually competitive with conventional electricity sources in an expanding list of geographic regions. Solar photovoltaics is now the third most important renewable energy source in terms of installed capacity in the world, right after hydro- and wind power. The long downward trend in retail PV prices continued in 2013, with a more pronounced reduction in Europe than in the USA [62]. Long term trends in retail prices are driven by the global supply/demand balance, by cuts in production costs and by changes in the government incentive schemes that stimulate demand. With sharp cutbacks in the European TiF schemes, the future evolution of prices is less obvious, although a further decrease is predicted. An additional factor to reduce retail module prices has been the concentration of productions in Taiwan and China [63]. Currently, solar module prices are below €1.50 per Wp, with the lowest prices quoted for a multicrystalline silicon solar module at €0.78/Wp (Germany). The lowest retail price for a monocrystalline silicon module is €0.81/Wp. The lowest thin-film module price is €0.62/Wp. As a general rule, it is expected that thin-film modules will sell cheaper than the crystalline silicon modules. The module cost is about 40 to 50% of the total installed cost of a PV system, bringing the total price of the installed multicrystalline silicon module to well below 2€/Wp in Europe [33, 64].

**Figure 5:**
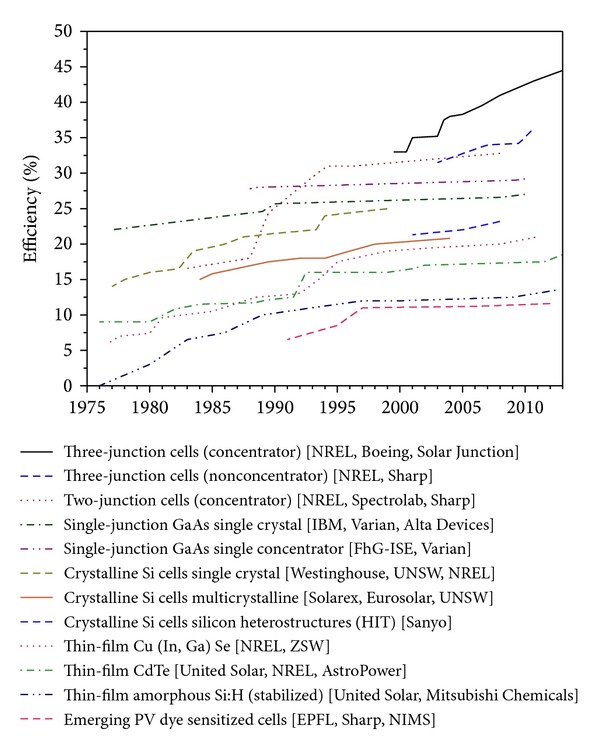
PV efficiency versus year of development [examples of developers]. Three-junction cells (concentrator) [NREL, Boeing, Solar Junction], three-junction cells (nonconcentrator) [NREL, Sharp], two-junction cells (concentrator) [NREL, Spectrolab, Sharp], single-junction GaAs single crystal [IBM, Varian, Alta Devices], single-junction GaAs single concentrator [FhG-ISE, Varian], crystalline Si cells single crystal [Westinghouse, UNSW, NREL], crystalline Si cells multicrystalline [Solarex, Eurosolar, UNSW], crystalline Si cells silicon heterostructures (HIT) [Sanyo], thin-film Cu (In, Ga) Se [NREL, ZSW], thin-film CdTe [United Solar, NREL, AstroPower], thin-film amorphous Si:H (stabilized) [United Solar, Mitsubishi Chemicals], and emerging PV dye sensitized cells [EPFL, Sharp, NIMS].

Conventional solar cells, made out of monocrystalline silicon, are the most commonly used in the market today. With enormous research, they have reached a high efficiency of 24.7% under standard test condition (STC). Sun power announced a 20.4% full panel efficiency which is a record efficiency as determined by measurements done by National Renewable Energy Laboratory [65]. Solar Press (UK) is working on sputtering technologies to achieve an even higher efficiency (up to 40%) [22]. Researchers are moreover developing other types of solar cells, such as the polycrystalline silicon solar cell and the thin-film solar cell. Thin-film cells are created by depositing thin layers of photovoltaic material on glass or stainless steel (SS) substrates. The advantages of this technology lie in that the thickness of the deposited layer which is normally less than 10 *μ*m (compared to several hundred *μ*m thickness for other PV cells) allows the creation of flexible PV modules, while reducing the costs of the semiconductor material. The current disadvantage of the thin-film PV results from a limited efficiency (less photovoltaic material present). The evolution of efficiencies of the different systems is illustrated in Figure 5, adapted from NREL [65].

A few typical examples of the different system developers are included in the caption of the figure.

The figure clearly illustrates that efficiencies of 20 to 25% are realistic for today's new PV modules, with higher efficiencies only proven on small scale at present.

Additionally, a major development involves the implementation of sun tracking systems, known to improve the PV efficiency by about 50% in summer to 20% in winter [66–68]. Such a tracking system is certainly economically justified for medium and large scale PV installations.

## 5. Comparison of Model-Predicted and Industrial Electricity Generation

**Figure 6:**
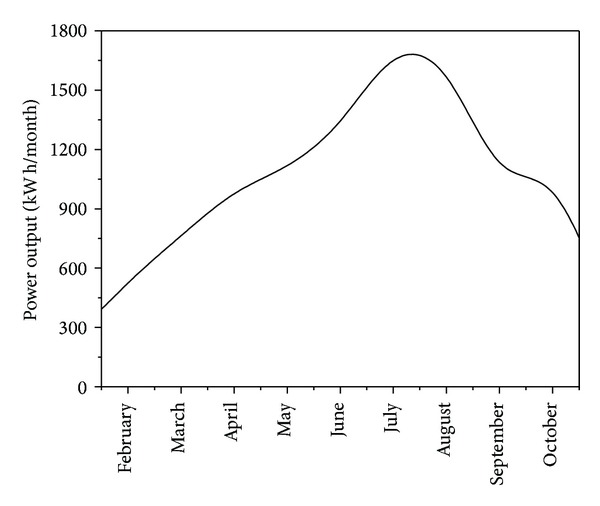
The power output of the solar panels in Arendonk (Feb 2011–Oct 2012).

In order to compare model predictions with real-scale data, results of a PV system in Arendonk (Belgium) were collected. The system includes 35 PV panels, inclined at 30 to 40° and representing a total surface area of 57.06 m². Measured power output is represented in Figure 6. Months from November to January are not included, since the production is low.

Having calculated the monthly and daily levels of solar irradiation by the approach of Zhang et al. [4], its impact on the power yield of the power plant can be assessed in order to define an overall productivity of the PV panels (including PV efficiency, downtime for maintenance, and inverter losses). The productivity is defined by the ratio of the electricity output (kW/m²) and the solar input (kW/m²).

**Figure 7:**
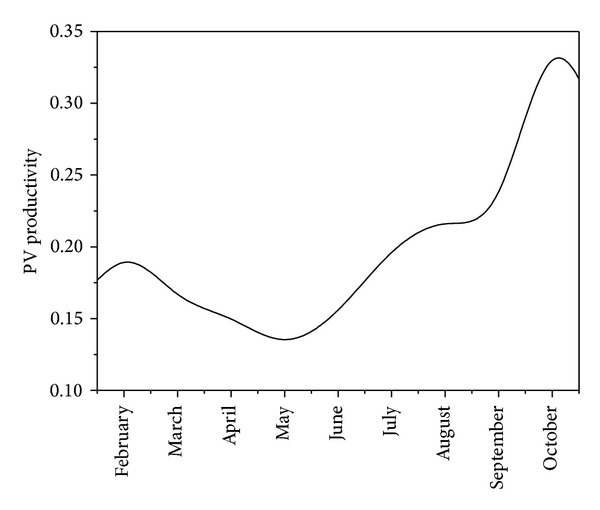
The productivity of solar panels in Arendonk (Belgium, Feb 2012–Oct 2012).

Results of the comparison are illustrated in Figure 7.

The productivity varies around 15 to 20% in the early months of the year, reaching values in excess of 20% during the summer and early-Autumn periods. The higher productivity in summer is a result of the higher solar irradiation levels in the summer months. The very high values obtained in September and October are better than expected, and the result of very clear days in these months of 2012 is with limited amounts of rainfall and lower average daytime temperatures with the associated reduction of thermal losses of the modules.

In general, to predict the PV output at any location, the procedure should be as follows:

- solar irradiation fluxes are calculated on hourly bases;
- the total energy flux absorbed by the PV field is calculated;
- the expected nominal capacity of the selected PV plant is calculated based upon the PV efficiencies of Figure 5, reduced by about 10% for inverter and maintenance losses.

## 6. Predictions of the Levelized Cost of Energy (LCOE)

A further demonstration of the PV potential for Europe is provided by a preliminary levelized cost of energy (LCOE) calculation. LCOE is usually expressed in US$/kWh or €/kWh.

LCOE is the price at which electricity must be generated from a specific source to break even over the lifetime of the project. It is economic assessment of the cost of the energy-generating system including all the costs over its lifetime: initial investment, operations and maintenance, cost of fuel, and cost of capital and is very useful in calculating the costs of generation from different sources.

The LCOE is calculated as the ratio of annualized investment cost (AIC, in US$ or €) and the annual electricity generation (*E*_an_, in kWh/year):

(3) $$\begin{array}{c} \text{LCOE}=\frac{\sum_{t=1}^{n}\left(\text{TICVC}/{\left(1+r\right)}^{t}\right)}{\sum_{t=1}^{n}\left(E_{\text{an}}/{\left(1+r\right)}^{t}\right)}, \end{array}$$

with LCOE, Levelized Energy Cost, as average lifetime levelized electricity generation cost; TICVC, the total of investment and variable costs of the complete process, including investment, operations and maintenance expenditures (in US$ or €); *E*_an_, the annual electricity generation (in kWh/year); *r*, the discount rate, taken at 10 %; *n*, the lifespan of the plant in years, taken as 20 years (the normal period of guarantee provided by the suppliers); and *t*, the year under consideration, from 1 to *n*.

Calculated values are given in Table 3.

The values presented in Table 3 clearly demonstrate that at current investment costs and efficiencies, as described in Section 4, LCOE-values are equivalent or lower than current household grid prices in most European countries, where values for private consumers range from 0.18 €/kWh (UK) to 0.25 €/kWh (Belgium). The spot electricity price received by the generators in the manner is however about 40% below the household prices (that include VAT and other taxes, including the renewable energy subsidy cost). Compared to spot electricity prices, PV has not reached grid party yet. With progressively decreasing or abandoned FiTs, grid party with household prices is only achieved at the lowest values of TICVC. Within the past and fast evolution of PV technology, it is clear that FiTs are difficult to set correctly and have been set too high in similar cases.

## 7. Conclusions

The present paper briefly reviewed the mechanisms, advantages and evolution of FiT. Although feed-in-tariff (FiT) mechanisms have been important in boosting renewable energy in Europe, the paper demonstrates that FiTs can be considerably reduced or even be eliminated in the near future in view of the predicted LCOE-values of current PV installations, ranging from 0.149 to 0.313 €/kWh, as function of the overall process efficiency and cost, and comparable with the current price of electricity supplied by the network.

Developments in PV efficiencies and costs are significant and increase the potential of PV applications in Europe and elsewhere.

A literature-based model predicts the solar irradiation on a monthly, daily, and hourly basis. Together with a performance factor, PV installations can be designed for a required power output. The annual average performance factor in a Belgian 57 m² PV unit was determined at about 20%, with higher values expected in more southern countries.

The paper provides design values for the amount of electricity generated by a PV system at any location and at a given efficiency.

## Figures and Tables

**Table 1:** Selected locations.

Location	Latitude	Longitude
Paris	48.8742°N,	2.3470°E
Brussels	50.8411°N,	4.3564°E
Frankfurt	50.1167°N,	8.6833°E
Seville	37.3833°N,	5.9833°W

**Table 2:** Weather conditions in the different locations in 2012 [23].

Location	Altitude (m)	Annual average wind velocity (km/h)	Annual average *T* (°C)	Annual precipitation (mm)
Paris	66	11.9	11.8	637.45
Brussels	55	13.2	10.4	767.54
Frankfurt	48	10.7	8.3	574.53
Seville	34	9.0	18.7	324.13

**Table 3:** Calculated LCOE, in €/kWh for different total investment cost and PV efficiency.

		LCOE (€/kWh)			
TICVC [(€)/Wp)]		Efficiency (%)			
		18	20	26	30
Investment	4 €	0.313	0.311	0.303	0.298
	3 €	0.235	0.233	0.227	0.224
	2 €	0.157	0.155	0.152	0.149
